# Standing horse posture: a longer stance is more stable

**DOI:** 10.1242/bio.059139

**Published:** 2022-05-12

**Authors:** Karen Gellman, Andy Ruina

**Affiliations:** 1 Maximum Horsepower Research, Ithaca, NY, USA; 2 Mechanical Engineering, Cornell University, Ithaca, NY 14853, USA

**Keywords:** Horse, Posture, Balance, Equilibrium, Stability, Mechanism

## Abstract

Horses stand for most of each day. Although they can use various leg configurations (postures), they usually stand with vertical legs. Why? We addressed this question with a 2D quasi-static model having three rigid parts: a trunk, massless fore-limbs and massless rear limbs, with hinges at the shoulders, hips, and hooves. The postural parameter we varied was ℓ_g_, the distance between the hooves. For a given ℓ_g_, statics finds an equilibrium configuration which, with no muscle stabilization (i.e. using minimal effort) is unstable. We assume a horse uses that configuration. To measure the neuromuscular effort needed to stabilize this equilibrium, we added springs at the shoulder and hip; the larger the springs needed to stabilize the model (*k*_min_), the more neuromuscular effort needed to stabilize the posture. A canted-in posture (small ℓ_g_), observed habitually in some domestic horses, needs about twice the spring stiffness (representing twice the effort) as is needed with vertical or slightly splayed-out (large ℓ_g_) legs. This relationship of posture and stability might explain the prevalence of vertical or slightly splayed-out legs in wild and healthy domestic horses and leaves as a puzzle why some horses stand canted-in.

## Abstract (precis)

**Summary:** Without stabilizing muscles, a standing horse is unstable. The bigger the spacing between fore and hind hooves, the less muscular effort needed for stabilization.

## INTRODUCTION

The assumption that horses tend to minimize metabolic cost predicts some aspects of horse locomotion, like gait transition speeds (Heglund et al., 1982; Gatesy and Biewener, 1991; Kram and Taylor, 1990; Hoyt et al., 2006). Perhaps minimization of energy, or of some other measure of effort, could explain other horse activities. While the metabolic costs associated with locomotion are obvious, it also takes some effort to stand still, and horses typically stand for 22–23 hours a day – eating, socializing and sleeping. This paper uses a simple model to explore the plausible idea that horses choose ways of standing that are relatively easy for them.

### Studies of horse posture

There are few studies of standing horse posture. Textbooks (e.g. Baxter et al., 2020), incorrectly, we believe, describe some postures as ‘conformations’, categorizing these postures as built-in physical features of the horse's body rather than as outputs of neuromuscular control. Horse postural sway has been characterized with stabilograms (trajectories of the net center of pressure) (Clayton et al., 2003; Clayton et al., 2013). As with humans (Kingma et al., 2011), the clinical utility of using stabilograms to measure postural competence is questionable; smaller sway may or may not be correlated with more robust standing. Lesimple, Fureix et al. (Fureix et al., 2011; Lesimple et al., 2012), studying spinal contours in standing horses, found that a horse with high head height, relative to back height, tended to be in stress or pain. Finally, physiotherapists found a correlation between equine thoracolumbar spinal contours and back pain (Shakeshaft and Tabor, 2020; Tabor et al., 2019).

**Fig. 1.**
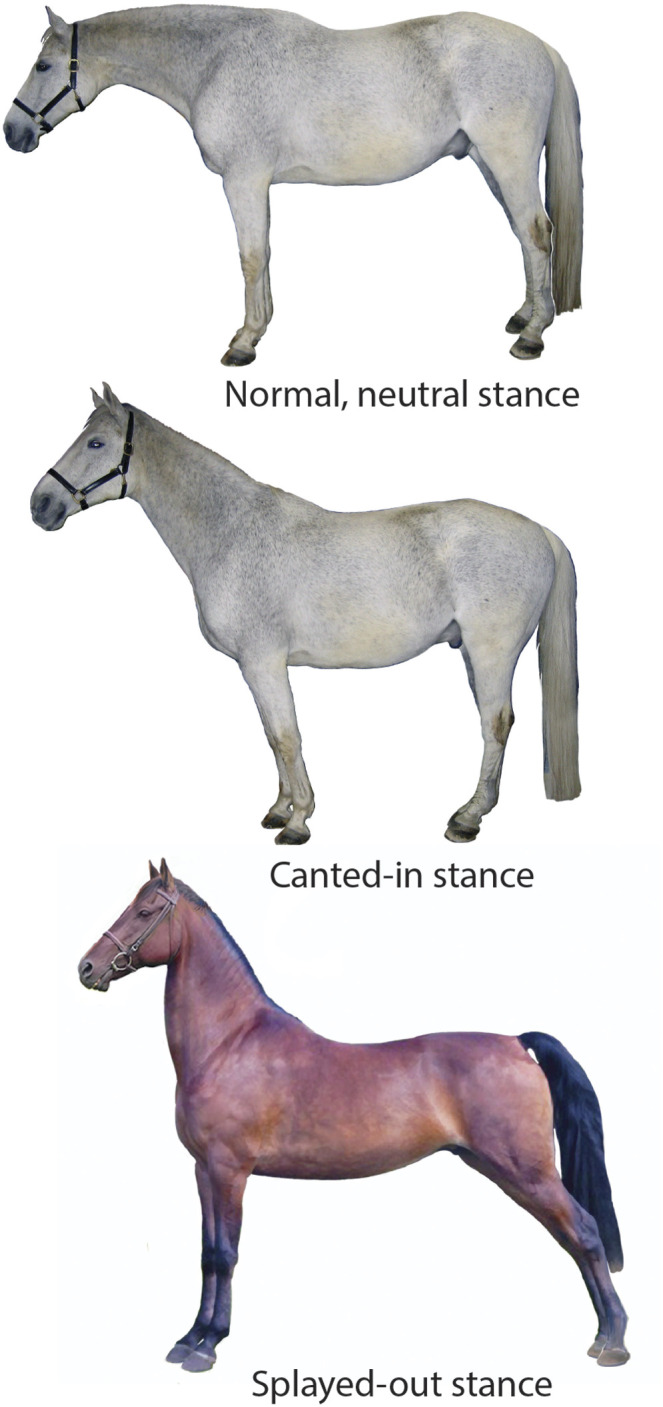
**Horse postures. Top:** *Normal*, neutral horse posture, legs are approximately vertical; **Center:** *Canted-in*, the fore hooves and rear hooves are closer together**. Bottom:** *Splayed-out*; the fore and rear hooves are relatively spread. In our model, large hoof-to-hoof spacing ℓ_***g***_ is splayed-out and small ℓ_***g***_ is canted-in.

We observe three categories of postural choice: vertical, splayed-out or canted-in (see Fig. 1).

### Vertical legs

Feral horses living in a natural unconfined environment, as well as most appropriately managed domestic horses, most often stand with visually vertical limbs. There are various reasons that long-legged animals might choose to stand with vertical, as opposed to canted-in or splayed-out, legs:

1. The compression force in the legs is slightly reduced by having vertical, as opposed to sloped, legs (see end of Section *Finding horse equilibrium postures*);
2. In locomotion, the range of leg motion is roughly centered at vertical. Protraction and retraction of the limbs are approximately symmetric relative to vertical. So, neither agonist nor antagonist muscles at the shoulder or hip are near their maximum (possibly uncomfortable) lengths when the legs are vertical.
3. The peak vertical ground reaction forces in running gaits occur when the legs are near to vertical, so the bones, muscles, tendons and ligaments are configured for carrying loads with vertical legs. Or, perhaps, standing with vertical legs might help align the internal structures for the high running-leg loads;
4. Horses are prey animals; a horse with vertical legs is perhaps most ready to escape in any direction.

Yet, horses do sometimes choose splayed-out or canted-in postures.

### Splayed-out posture

**Fig. 2.**
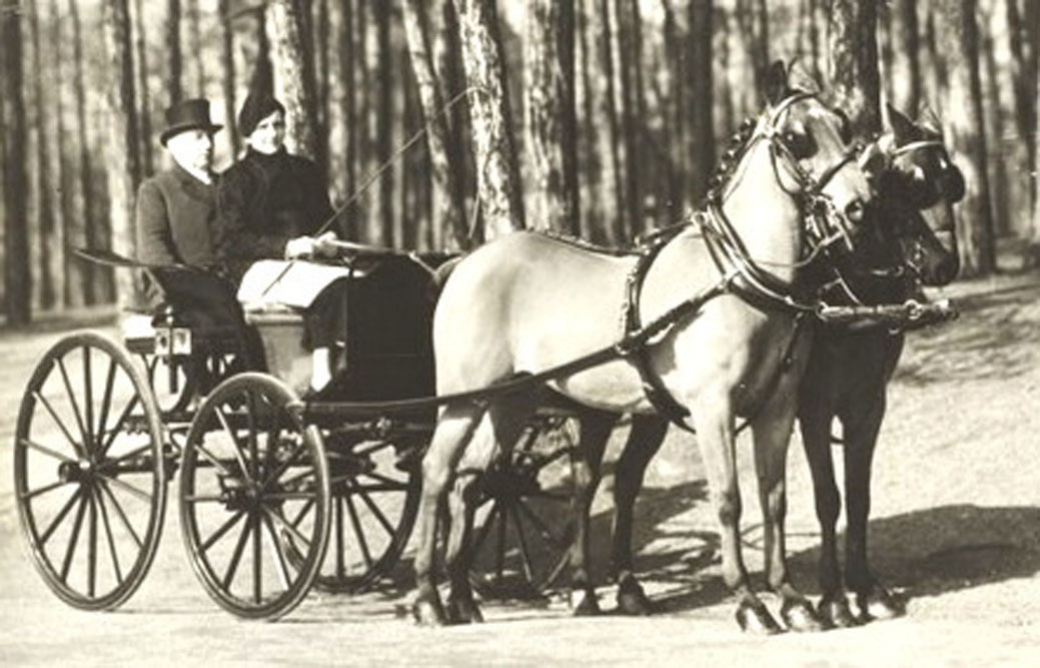
**‘Parked’ horses have a splayed-out posture.** Crop from ‘Horse and Carriage’ by Dora Maar from Cleveland Museum of Art (circa 1931–1936). ©2021 Artists Rights Society (ARS), New York / ADAGP, Paris. All reproductions of this work are excluded from the CC: BY License.

In long-legged vertebrates, splayed-out postures are often seen when greater stability is useful. These situations can be functional, like a weight-lifter's wide stance or people bracing while standing on a moving bus. Or, they could be compensation for neural impairments (Steinberg et al., 2000; Thompson, 2012; Mayhew et al., 2013; Bunn et al., 2013). We see horses adopting a spread posture when carrying a heavy rider, when holding a heavy cart stationary (‘parked’), when in the late stages of pregnancy, or in the first hours of standing after birth. The horse-show term ‘park’ refers to the wide front to back stance traditionally used by carriage horses to hold a vehicle motionless (see Fig. 2). The intuitive idea, which we buttress with our model here, is that a splayed-out posture is easier to stabilize.

### Canted-in posture

On the other hand, canted-in postures, in which horses stand with their hooves relatively close together, are seen in horses with Equine Motor Neuron Disease (Divers et al., 1997; Valentine et al., 1994) and in some horses with chronic performance deficits (J.M. Shoemaker, DVM – personal communication). In contrast to the stability gain from splayed-out postures, the possible reasons some horses choose a canted-in posture are less obvious: Perhaps,

1. The horse has distorted proprioceptive signals, resulting in a distorted postural output that is canted-in;
2. The horse has an impairment of postural control or motor output such that it is unable to stand with vertical or splayed-out legs;
3. The canted-in posture allows the horse to ‘lean’ on ligaments or bones in ways that (while possibly painful or injurious) might relieve the burden on fatigued or impaired muscles;
4. One model of human stance, which takes account of neural delays, finds that the range of stabilizing control gains is smaller for a splayed-out posture (Scrivens et al., 2006). Applying that model to a standing horse, we would presumably find that a horse, like a human, would also find a wider range of stabilizing gains with a canted-in posture (see Section *Model Choices B* for further discussion).

### Shifting weight

It might seem plausible that a canted-in posture, with front and rear hooves relatively near to each other, would allow the horse to more easily alter its load distribution between front and rear limbs, with only minor variations in posture. However, preliminary data using a force plate under each hoof indicates that for all postures, including canted-in postures, horses hold about 60% of body weight on forelimbs and 40% on rear limbs with little variation over time (Gellman, Shoemaker and Reese, unpublished data).

### Postural choice effect on stability

Our primary question is this: how does a horse's choice of leg splay (vertical versus splayed-out versus canted-in) affect the difficulty of maintaining posture? For a given leg splay, we assume the horse finds a minimum-effort configuration. Then, we quantify the (in)stability of that configuration.

### Related stability-of-mechanism models

Our approach is similar to that used by others for stability of grasp, and for lateral (side-to-side) stability of standing people and cats. The common idea is that these systems, idealized as a mechanisms (rigid bars connected with hinges), still have ways to move, even while respecting the mechanism constraints. But the mechanism must be stable against those possible ways to move. For example, a grasp, i.e. fingers pinching an object, must be stable for the finger configuration to be maintained. The muscles and tendons achieve this stability either by the intrinsic stiffness of muscles used for the task, or by stiffening the joints, either literally, using the stiffness of additionally contracted muscles, or possibly with fast feedback (e.g. Sharma and Venkadesan, 2022; Rancourt and Hogan, 2001). The extra stiffness needed for stabilization is *k*_min_. In Bunderson et al. (2008) this idea is applied to a 3D model of a cat leg. The sagittal-plane study of human standing in De Groote et al. (2017) demonstrates that standing stability is achieved, in part, by intrinsic (initial short-time mechanical response, or ‘short-range’) muscle stiffness. Other studies consider stabilization of a four-bar linkage model of standing, and some explicitly include the time delays (due to transmission from sensors to the spine or brain, neural processing, transmission back to the muscle and muscle activation rise time; Scrivens et al., 2006; Bingham et al., 2011; Goodworth et al., 2014).

### How to quantify instability

There is no best way to quantify the ‘difficulty of maintaining a configuration’. The appropriate measure of difficulty depends on the nature of the employed control system. In particular, the torques used to stabilize an equilibrium posture may come from tonic contractions or co-contractions (see Section *Model choices B*), from proportional feedback with delays (e.g. Goodworth et al., 2014), or from intermittent feedback based on an internal model (Loram et al., 2005b; Morasso et al., 2015) and each of these is challenged by different aspects of the mechanism. We seek the simplest reasonable measure.

### The minimum stabilizing spring *k*_min_

We quantify the instability challenge of a posture by the minimum stiffness (*k*_min_) of shoulder and hip springs that, if added, could minimally stabilize an equilibrium posture. Use of *k*_min_ as a proxy for neuromuscular control effort may be regarded as measuring the difficulty of maintaining, or nearly maintaining, balance with tonic contractions alone. Or, more generally, and vaguely, if active neural feedback control is involved, *k*_min_ might be interpreted as representative of the integrated effort of tonic and feedback control mechanisms.

## RESULTS

### Outline of the calculations

Using a three-link model, we consider two statics features:

1. **Geometry of muscle-torque-free equilibrium.** For a given foot spacing ℓ_g_, basic statics finds those postures for which the linkage carries the whole load without any joint torques, assuming a perfect model and no disturbances (see Section *Finding horse equilibrium postures*). In these joint-torque-free postures the ‘horse’ (our model horse) and all of its parts obey the laws of static equilibrium (forces and moments ‘balance’ on all of the parts). For symmetric models, as in Bingham et al. (2011) and Goodworth et al. (2014), this equilibrium posture is symmetrical so is found without calculation. Because a horse (and our model) is not front-back symmetric, finding equilibrium postures requires a statics calculation.
2. **Stability of equilibrium.** As for a pendulum (described in detail below), an equilibrium posture, as found above, can be stable or unstable. An unstable equilibrium posture will, if disturbed slightly, tend to deviate progressively. In contrast, a stable equilibrium posture is one that, if slightly disturbed, will spontaneously tend to recover. Here, we want to quantify the degree of instability of an unstable posture. Our proxy for the neuromuscular effort needed for stabilization is the amount of joint torque needed to bring the horse back to equilibrium after a given small disturbance, assumed to be proportional to the amount of disturbance. We quantify instability by the stiffness of the springs *k*_min_ at the shoulder and hip needed to achieve stability. Note, this differs from the quantification of instability used in Scrivens et al. (2006) and Bingham et al. (2011) (see Section *Model choices B*).

Overall, there are three nested problems: (1) A horse chooses a leg spacing; (2) given that spacing, it chooses leg and back angles; (3) given that spacing and the leg angles, it uses some stabilization strategy. For any (1) leg spacing, there is some (2) posture and (3) control strategy that minimizes effort. We assume that our model horse's choice of leg spacing would be informed by the consequent effort of stabilizing the associated equilibrium posture.

Our calculation can best be understood by considering it in the context of a simpler system, a pendulum. An inverted pendulum is in equilibrium if it is exactly upright. This is an unstable equilibrium that can be stabilized with a sufficiently stiff torsional spring *k*_min_. The pendulum analogy is presented in detail at the beginning of Materials and Methods.

#### The torque-less equilibrium postures

These postures, with no muscle torques at the shoulder and hip joints, have a simple geometric interpretation (Fig. 5). Consider a vertical line through the center of mass; a line defined by the front hooves and shoulder; and a line defined by the rear hooves and hip. Because the legs are ‘two-force objects’ and the body is a ‘three-force object’ these three lines are either parallel (Fig. 5a), or they intersect above or below the horse at C (Fig. 5b and c, respectively). These postures, if exact, do not need joint torques to be maintained. Thus, we expect the postures that horses adopt would be close to these. For example, if the legs are parallel, then they should be vertical (not slanted) with respect to the ground. A horse with parallel slanted legs is not in a torque-free equilibrium posture; hip and shoulder torques would be required to hold that position.

### Quantification of instability

As noted, for a standing horse, torque-less equilibrium postures that are close to plausible real-life horse postures are all unstable. That is, absent corrective torques, the equilibrium postures are postures in which small deviations from equilibrium would tend to grow rather than be naturally restored. For all of the equilibrium postures of interest, the center of mass height is at a (local) maximum. This unstable feature of equilibrium occurs whether point C, the leg-lines intersection point, is above or below the horse. In the model here, we add springs at the hip and shoulder and find *k*_min_, the smallest spring that stabilizes the muscle-free equilibrium postures. Our basic modeling postulate is this:

A posture with a bigger *k*_min_ is more unstable.

**Fig. 3.**
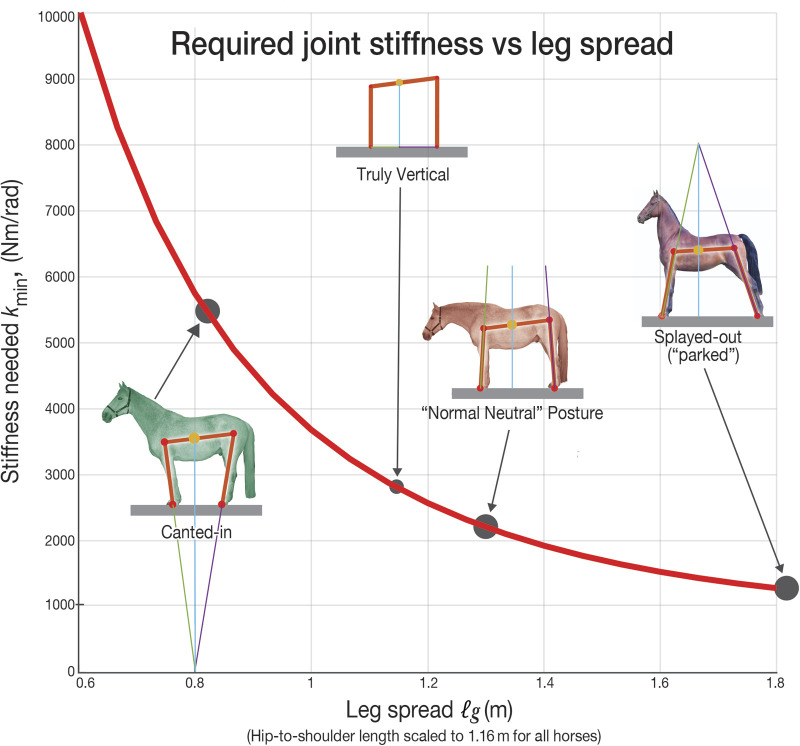
**Stiffness versus Leg Splay.** For a given horse geometry (leg lengths, back length, location of CoM) and for a given splay ℓ_*g*_ (distance between fore and rear hooves) the ‘horse’ (modeled as a linkage) has an equilibrium configuration satisfying the statics two-force-object rules for the legs and the statics three-force-object rules for the horse (see Fig. 6 and Fig. 5). The size *k*_min_ of the minimum stabilizing springs at hip and shoulder is plotted as a function of leg splay ℓ_g_. The central results of this paper are the differences between *k*_min_ for the normal neutral posture (NNP) and for the splayed-out and canted-in postures. To be stable, the canted-in posture needs a stiffness (*k*_min_≈5400 Nm/rad) that is more than twice that needed for NNP (*k*_min_≈2100 Nm/rad). The splayed-out posture is even more intrinsically stable than normal neutral posture (NNP) in that it needs only about 60% of the *k*_min_ required by NNP.

The central result of the calculations is shown in Fig. 3:

A horse, when legs are canted-in (legs closer together) needs much larger corrective springs *k*_min_, by more than a factor of 2, than when legs are vertical or slightly splayed-out.

As the corrective springs are a proxy for neuro-muscular effort, it follows that it takes less neuro-muscular effort for horses to stand with a normal neutral posture than with a canted-in posture.

A final statics result is that, within the commonly observed range of horse postures, the compressive forces in the legs, while minimized by vertical legs, are only slightly (we think negligibly) increased with canted-in or splayed-out legs (see Section *Modeling choices A*).

## DISCUSSION

In normal, sound horses the most common posture is standing with the metacarpal and metatarsal bones (the lower leg bones) visually vertical. In this posture the legs are slightly splayed-out with reference to the shoulder/hip to hoof angle, as seen in Fig. 3.

We term this normal neutral posture (NNP), normal in that it is most common in healthy and feral horses and neutral in that it equally allows leg movement in any direction. Our model predicts less passive instability in splayed-out postures, thus requiring less tonic contraction, and correspondingly less effort, to stabilize. This possibly explains why horses needing extra stability often display splayed-out postures, and why we rarely observe persistent canted-in postures in normal healthy horses.

Like horses, humans adopt a wider stance when lifting heavy weights or standing on unstable surfaces. Why do humans and quadrupeds not always use a more spread stance? Stability could be only one of several situation-dependent goals. In Fig. 3, the change in *k*_min_ between NNP (slightly splayed) and more obviously splayed-out posture is relatively small. Perhaps the various benefits of nearly vertical legs, mentioned in the introduction, outweigh the relatively small stability-benefit gains of a still-wider splayed-out stance.

### Why are canted-in postures ever seen in horses (or people)?

By our calculations, canted-in postures are far less stable than vertical or splayed-out postures. Assuming our calculations are relevant, and that maintaining stability with minimal effort is a high priority for the horse, how can we explain that some horses habitually use a canted-in posture? Narrow-stance postures seem to be associated with impairment of either sensory input, neural processing, or motor output concerning postural control. For example, in

1. **Parkinson's disease (PD)** patients (humans) have a characteristic collapsed standing posture that includes having a narrow stance. PD is a degenerative disorder of the human central nervous system (Kim et al., 2009) in which patients have lost dopaminergic neurons in the midbrain, a critical region for postural control, resulting in a dysfunction of central postural processing.
2. **Equine Motor Neuron Disease (EMND)** is a condition for which canted-in limb posture is pathognomonic (characteristic) in horses. In this degenerative neuropathy, similar to ALS in humans, there is cell death and consequent denervation atrophy in postural muscles (Valentine et al., 1994). EMND patients habitually display extreme canted-in postures, fatigue quickly, and spend much time lying down (Divers et al., 1997). This is primarily a defect in the postural motor output. Perhaps EMND patients are also ‘leaning’ on passive ligaments, as described in the Introduction's list of canted-in hypotheses. This could be investigated by measuring EMG activity during this stance. However, since the recognition of EMND's nutritional etiology (selenium/vitamin E deficiency), few clinical cases have presented in recent years.
3. **Abnormal Compensatory Posture (ACP)**, a narrow stance seen in some domesticated horses, appears to be caused by structural distortions in some critical mechanoreceptor-rich anatomic regions (the hooves, the stomatognathic system, and the upper cervical area) due to practices associated with domestication. In these horses, the inaccurate proprioceptive signals (sensory input) are altering neural processing and motor output. Horses habitually standing with canted-in posture often develop a predictable set of musculoskeletal problems, including: navicular syndrome, negative palmar/plantar angles, digital cushion degeneration, hock and stifle osteoarthritis, sacroiliac fixation or hypermobility, back pain, ‘kissing spines’, neck pain, and more (J.M. Shoemaker DVM – personal communication). The postures of these horses revert to a visually vertical stance (NNP) when these structural and functional distortions are corrected (Gellman, Shoemaker, and Reese, unpublished data). Fig. 1 shows the same horse, on the same day, before (canted-in) and after (normal) a manual therapy intervention in the upper cervical region.

### Exhausted at ‘the end of the trail’

A famous sculpture of a horse and rider, ‘The End of the Trail’, by J. E. Fraser, shows a Native American man slumped over a presumably exhausted horse with an extremely canted-in posture (Fraser, 1918). However, we do not have any other evidence, even anecdotal, that a canted-in posture is adopted by exhausted horses. For example, this is not seen at the end of modern horse races. Historically, horses raced for far longer distances, or pulled carts for long hours in transportation or military operations. Possibly, modern recreational use of horses does not result in the kind of extreme exhaustion depicted in that sculpture. Possibly the artist used a horse model affected by one of the mechanisms listed above. Or the depiction may just be artistic license.

### Intuitive explanation of stability results

The basic result, that a splayed-out posture is more stable (by our measure), can be predicted qualitatively without need for detailed calculations. For the torque at a joint to have effect on the whole-mechanism motion, the whole-mechanism motion must involve changes in that joint angle. Imagine a horse that is so canted-in that the front and rear hooves nearly touch. Then, if that horse rocks forward (or backward), still keeping its feet on the ground, there is significant falling even though there is almost no change in the shoulder or hip angles (the horse falls almost like a triangle rocking on its lower vertex). Thus, for that extremely canted-in posture, springs at the shoulder or hip joints would have almost no effect (have almost no righting torque, cause almost no change in potential energy, do almost no work), requiring bigger stabilizing springs. When the legs are more spread, the joint angle changes are bigger for a given rocking. Bigger joint-angle changes have a dual effect: bigger angle changes with a given amount of falling cause bigger rotation-induced spring torques; and, due to the reciprocal nature of mechanical advantage, also, have a bigger mechanical advantage (i.e. bigger effect on the mechanism for a given spring torque). Meanwhile, the curvature of the gravitational potential energy near equilibrium, as a function of leg splay, is slight (the circular arc of CoM motion when the legs are parallel is similar to the near-circular arc when the legs fully canted-in). So, due to the enhanced utility of the springs in splayed-out posture, the splayed-out posture is ‘more’ stable by our measures.

### Comparison with calculations in Bingham et al.

As noted, Bingham et al. (2011) use a mechanical model, similar to ours, but come to the opposite conclusion. They predict that a narrow-stance (canted-in, small ℓ_g_) posture is ‘more stable’ whereas we find it is less stable. Besides a difference in mechanical parameters, there are two differences between our model and theirs: (1) they include delays, we do not; and (2) they quantify difficulty of control by the smallness of the range of gains which is stabilizing, independent of gain size. In contrast we quantify difficulty by the size of the needed gains, independent of the range of stabilizing gains. Their stability criterion is not applicable to our model because we ignore time delays, making our range of stabilizing gains semi-infinite for all postures.

Bingham et al. argue that narrow stance might benefit human PD patients because, in their model, the range of feedback gains that stabilize a narrow stance is greater than the range of gains that can stabilize a wider stance. Thus, they claim, finding a successful stabilizing gain may be easier for a narrow rather than for a wider stance [see Section *Model choices B* for further comparisons with Bingham et al. (2011)]. While their narrow-stance model seems to describe neuro-compromised PD patients, it does not align with most observable human behavior and experience, which is that we tend to adopt a wider stance when greater stability is needed.

### Implications for neuromuscular control: avoidance of the need for fast feedback

Depending upon the demands of the situation, instabilities of equilibrium postures can be more or less of a problem for the system (animal). For instance, stabilization with feedback may be impossible in a finger pinch because of neuro-muscular delays, sensor noise (Sharma and Venkadesan, 2022), and short characteristic instability times. For longer characteristic instability times, where feedback control is plausible, such feedback will necessitate fluctuating muscle forces, which adds extra metabolic cost (as seems to be the case with human standing; Loram and Lakie, 2002; Goodworth et al., 2014). Thus, animals may often seek, by some mixture of task choice, postural choice and tonic contractions, a non-feedback mechanism, namely some kind of tonic contraction, to obtain near-stabilization. Then, they only use feedback control for final stabilization and for controlling motion of the slowed system.

One could speculate that overall system design might also be driven to avoid instabilities that are fast, and thus difficult or expensive to control. This might have driven bicycle designs towards passive stability (https://ecommons.cornell.edu/handle/1813/22497) or driven the human skeleton to a configuration where passive walking is almost stable (e.g. McGeer 1990; Garcia et al., 2000).

### More fundamental modeling

As noted in the Introduction, a horse contends with three optimization problems: (1) how far to spread the hooves, ℓ_*g*_; (2) the joint angles to use as a target for control (the hip angle); and (3) the strategy of stabilization (tonic versus feedback). This paper has directly addressed the first two, and merely assumes that the third is largely based on tonic stabilization.

As suggested by Kuo (personal communication), in principle, we could (and should) construct a model for standing that includes metabolic costs for force and rate of change of force, sensor or actuator noise, and neural delays. Optimization of energy use in such a model might (we hypothesize) demonstrate the plausibility of there being metabolic benefits for strategies that use a mixture of tonic and feedback mechanisms of the general type that we have assumed in our modeling here and has been noted by others (e.g. Loram et al., 2005a; Sharma and Venkadesan, 2022).

### Implications for horses

Neither zoologists nor veterinarians have an accepted standard for ‘normal’ standing horse posture. We propose that visually vertical limbs (NNP) should be considered normal posture. Because standing is such a prevalent activity for horses, perhaps evaluation of habitual posture should be considered as part of clinical exams, and in developing management programs. The slightly-splayed-out NNP posture may represent a trade-off between the stability benefits of more extreme splay and, e.g. the quick-escape benefits of mostly vertical legs. On the other hand, sports injury, poor athletic performance, and chronic or recurring lameness are common sequelae in domestic horses who habitually display abnormal compensatory posture with canted-in limbs rather than normal neutral posture. Future studies will aim to characterize the anatomic distortions associated with ACP and track restoration of NNP through correcting these structures.

## MATERIALS AND METHODS

### Modeling choices, in brief

The following are some modeling considerations. These are discussed further in Section *Modeling choices A*.

**2D versus 3D.** For simplicity, and because it corresponds to the horse observations that motivated the study, we use a 2D sagittal plane (side view) model of a horse.
**Three-link mechanism.** We model the horse as having three rigid links: the rear-leg pair, the front-leg pair, the horse body and head. This is sometimes called ‘a 4-bar linkage’, counting the ground as the fourth link. We neglect the weights of the legs, joint friction, all soft-tissue deformation, deformation of the spine and relative rotation at all joints except the hips and shoulders.
**Base of support.** We do not consider the toppling of a horse, the tipping over due to the center of mass going outside the quadrilateral defined by the four hooves. Instead, we are considering the stability of the mechanism's shape.
**Leg compression force.** We do not consider the effect of posture on leg compression force. We think this effect is smaller than the stability effects.

### Pendulum analogy

**Fig. 4.**
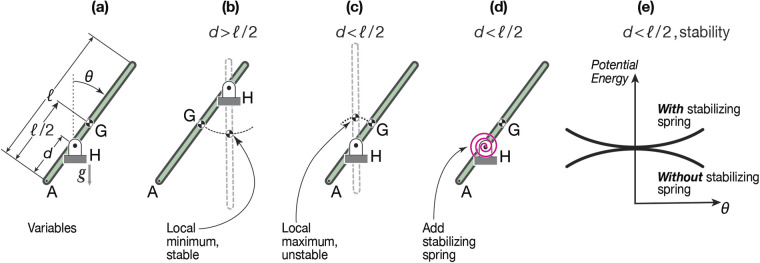
**Pendulum: Stability, and stability of equilibrium. (a)** The pendulum has its lower end at A, hinge at H, Center of Mass at G, and is tipped an angle ***θ*** from vertical; **(b)** CoM below the hinge is stable, equilibrium is at a potential energy minimum; **(c)** *d*<ℓ/2, CoM above the hinge is unstable, equilibrium is at a potential energy maximum; **(d)** a spring is added to the unstable configuration; **(e)** the unstable configuration is made stable by the addition of a spring, changing a potential energy maximum (bowl down, unstable) into a potential energy minimum (bowl up, stable).

A pendulum is a rigid stick with length ℓ that is connected to a fixed support by an ideal hinge at H (Fig. 4). In this example, mass is uniformly distributed on the stick. The average position of the pendulum's mass is at the center of mass (CoM or G). The hinge H and center of mass G are distances *d* and ℓ/2 from the lower end A, respectively. The hinge H does not resist rotation of the stick. However, there could be torque at the hinge due to some proxy for muscles (e.g. a motor or a torsional spring). In this analogy, ‘posture’ combines the distance *d* of the CoM G from the end (analogous to ℓ_*g*_ on the horse) and, secondarily the angle *θ* (analogous to the leg angles the horse chooses for a given ℓ_*g*_).

#### Non-equilibrium ‘posture’ for a pendulum

The tipped configurations in Fig. 4 are all not in equilibrium; they have *θ*≠0 and *θ*≠*π*. In these non-equilibrium configurations, the moments do not balance, and the stick tends to swing or fall down. If the stick is put in such a non-upright (non-equilibrium) configuration and the hinge is not at the center of mass (*d*≠ℓ/2) then: if the center of mass G is below the hinge (ℓ/2<*d*, Fig. 4b) the pendulum will swing down towards vertical; and if G is above the hinge (ℓ/2>*d*, Fig. 4c), it will fall away from upright towards the hanging down configuration.

#### Equilibrium ‘posture’ for a pendulum

The equilibrium configurations are those for which the stick is vertical (*θ*=0 or *θ*=*π*), shown dotted in Fig. 4b and c. [Aside: A special set of equilibrium configurations are when the hinge H is at the center of gravity G (*d*=ℓ/2) and the stick is in equilibrium at all angles (there is no analogue for this set of ‘neutral’ stability configurations in the horse model).] Assuming no disturbances, it takes no torque to hold the equilibrium configurations (*θ*=0 or *θ*=*π*). The pendulum equilibrium *θ* does not depend on the choice of *d*. For the horse model, however, which does not have front-back symmetry, the leg angles associated with equilibrium do depend on the hoof spacing ℓ_*g*_.

#### Stability of pendulum equilibrium

The vertical equilibrium postures, with the center of mass G below (*d*>ℓ/2) or above (*d*<ℓ/2) the hinge H, are intuitively quite different from each other. Actually, if the center of mass is above the hinge, in an ‘inverted pendulum’ configuration, many people have trouble accepting that this is an equilibrium position at all; in an experiment, if the center of mass is above the hinge, the stick would obviously fall. Nonetheless, if it was a perfect stick, placed perfectly vertically, and perfectly following the laws of classical mechanics, it would not fall. So, the position where the center of mass is directly above the hinge is, at least in the language of mechanics, ‘in equilibrium’; to keep that stick upright you do not need to apply a torque to the left, nor to the right. On the other hand, practically speaking, something has to be done to keep the stick from falling. That is, the upright configuration is an equilibrium posture, but it is an unstable equilibrium. It requires the addition of some stabilization torques to be maintained. Summarizing,

- **Equilibrium** postures can be held with no extra torque at the hinge, for the pendulum these are the vertical configurations;
- **Non-equilibrium** postures, tipped sticks, would require additional constant joint torques to hold those postures in equilibrium;
- **Stable equilibrium** postures are ones where, if disturbed from equilibrium a tiny amount, the system will tend to return towards that equilibrium with no muscular torques (dotted stick in Fig. 4b);
- **Unstable equilibrium** postures are ones where, if disturbed from equilibrium a tiny amount, the system will tend to exponentially deviate from the equilibrium (dotted stick in Fig. 4c). Unstable equilibrium postures need additional stabilizing torques to be maintained.

### Quantification of pendulum instability

The horse postures we are interested in are all, without additional muscle use for stabilization, unstable. So, we only need to consider the unstable, inverted pendulum (Fig. 4c). Having the hinge below the center of mass (*d*<ℓ/2) is like the horse being above the ground; having *d*>ℓ/2 would be analogous to a horse hanging by its hooves upside down from the ceiling (to be complete, depending on leg lengths and back length, there may also be non-physical cockeyed equilibrium postures that our model could exhibit, but which a real horse could not achieve).

#### Intuitive discussion of instability measures

Imagine balancing an upright upside-down broom on the open palm of your hand. You accomplish this by moving your hand around appropriately. Now imagine trying to similarly balance a pencil. In that context one would say that a pencil is more unstable than a broom; it is much harder for a person to balance a pencil. On the other hand, imagine a vertically upright 5 m (quite tall) ladder resting on the ground, but leaning against nothing. You are holding it upright. Then imagine a person climbing to the top, and you are still trying to balance the ladder. Clearly the ladder is harder to balance when there is a person on top and is, as measured by difficulty of balancing, more unstable.

For the pencil versus broomstick, the pencil is more unstable because it falls more quickly. For the ladder versus ladder with person up top, the heavy-top ladder is more unstable because for a given angle of tip it takes more force to keep it from falling.

More technically, for the pencil versus broomstick, initial falling occurs with exponential growth *θ*∼*e*^*λt*^. The pencil has a bigger eigenvalue *λ* than does the broomstick. For the ladder versus the heavy-topped ladder, we look at the minimum possible spring *k*_min_ at the hinge H that would stabilize the ladder. The needed spring *k*_min_ is bigger for the heavy-topped ladder. Thus eigenvalue *λ* and minimum corrective springs *k*_min_ are two different measures of instability. A third measure proposed by Bingham et al. (2011) is discussed in Section *Model choices B*.

#### Comparison of instability measures

The two measures (above) are quite different in their predictions of what systems are more or less stable. Imagine an inverted pendulum consisting of a massless rigid stick with length ℓ hinged at the bottom and a point mass *m* at the top. Consider three other sticks, one with twice the mass *m*, one with twice the length ℓ, and one on a planet with twice the gravity *g*:

1. Double *m*. This doubles the needed stabilizing spring *k*_min_ but has no effect on the eigenvalue *λ*. Thus, this would be a more unstable pendulum by the *k*_min_ measure and equally unstable by the *λ* measure.
2. Double ℓ. This slows the falling but increases the spring needed for stabilization. That is, the two measures of instability have opposite trends for pendulum length changes.
3. Double *g*. By both measures, *k*_min_ and *λ*, doubling *g* is more unstable. So, the measures agree about the relative stability.

That is, saying which physical system is more or less unstable than another depends on how instability is quantified. We assume here that the *k*_min_ measure is most relevant because perhaps, as per, e.g. Loram and Lakie's result for fore-aft balance of standing people (Loram and Lakie, 2002), the horse may use tonic means to almost stabilize. Using this strategy, the horse avoids being challenged by the characteristic time of instability being shorter than neural delays.

#### Potential energy: minimum versus maximum determines stability

First, consider a pendulum with gravity but no spring. The stick is in equilibrium if it is vertical. The vertical equilibrium is only stable if the center of mass G hangs below the hinge H (*d*>ℓ/2 in Fig. 4b). The vertical equilibrium is unstable if the center of mass is above the hinge (*d*<ℓ/2, Fig. 4c). For this system, the potential energy (PE) is weight times the height of the center of mass (relative to an arbitrary datum, say the pendulum hinge). Relative to nearby configurations (nearby values of *θ*), the vertical stick is at a locally minimum PE height (i.e. stable) if the center of mass G is below the hinge H. And it is at a locally maximum PE height (i.e. unstable) if G is above H.

#### Potential energy quantification of pendulum instability

The *k*_min_ approach to quantifying instability can also be understood using potential energy. We imagine that we have applied a corrective spring at H that tries to center the pendulum at *θ*=0. The total PE, (*E*_p_), is a sum of gravity (destabilizing) and spring (stabilizing) terms,
(1)

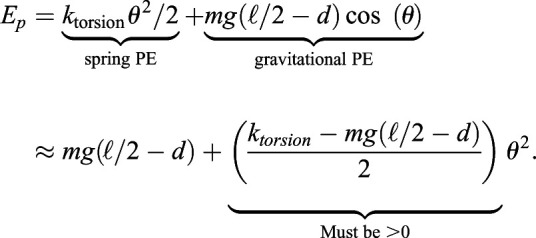
Note that ℓ/2−*d* is the distance along the stick that the center of mass G is above the hinge H. We used the small-angle approximation that cos *θ*≈1−*θ*^2^/2. For stability, we want *E*_p_ to be an upwards shaped bowl (concave up) function of *θ* with the equilibrium *θ*=0 being at a potential energy minimum. Thus we need the coefficient of *θ*^2^ to be positive (Fig. 4e). Such stability occurs if the torsional spring has a big-enough constant,
(2)

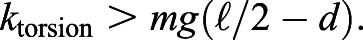
That is, the otherwise-unstable vertical pendulum is stabilized by the addition of a *k*_torsion_ satisfying the inequality above. With that big-enough spring, as *θ* is deviated from 0, the increase in spring energy will win over the decrease in gravitational potential energy. The more that the center of mass is above the hinge (the bigger the distance ℓ/2−*d*), the bigger the stabilizing spring *k*_min_ needed to make this otherwise unstable equilibrium posture stable.

#### Application of the pendulum analogy to the horse model

With the horse model, we similarly find an equilibrium posture, the stability of that equilibrium, and the minimum spring needed for stabilization. We assume that the horse chooses leg angles so as to be in equilibrium with no muscle torque, which is analogous to only considering stability of a vertical pendulum. The calculations for the horse model are more complicated, and in the horse model, the amount of spring needed depends on the posture (the spacing between the feet ℓ_*g*_) rather than the relative positions of G and H (the value of ℓ/2−*d*) for the pendulum.

### Finding horse equilibrium postures

Given a hoof spacing ℓ_*g*_ we assume the horse chooses a posture which minimizes muscle effort. A posture that is in equilibrium when there are no joint torques would be a minimal muscular effort posture. Such a posture is analogous to the vertically upright pendulum configuration. For each leg spread ℓ_*g*_ we find this posture using elementary statics.

#### Features of the relevant free body diagrams

**Fig. 5.**
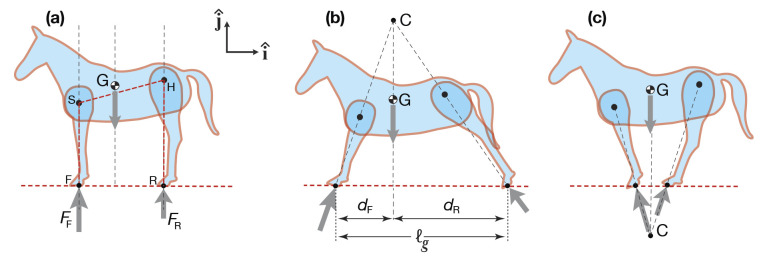
**Equilibrium postures that do not need any muscle torques. (a)** Vertical legs; **(b)** Splayed-out; and **(c)** Canted-in. In our 2D model, a whole horse has three forces acting on it: (1) gravity at the CoM; (2) the ground reaction force on the fore feet; and (3) the ground reaction force on the rear feet. Static equilibrium of the whole horse, a ‘three-force object’, demands that these ground forces are either **(a)** parallel and vertical; or **(b)** they intersect directly above the CoM at C; or **(c)** they intersect directly below the CoM at C (see text for reasoning). The joint springs that would be needed to stabilize these unstable equilibria are the core of this paper and are considered in the Section *Calculations of stability* and Fig. 3.

A free-body diagram is a sketch of the isolated system (free body) and of all of the external (from outside the system) forces and moments (torques) on it. We consider three free-body diagrams:

(1) The whole horse including body, head, tail and legs (Fig. 5);
(2) The fore-legs, and
(3) The hind-legs.

**Fig. 6.**
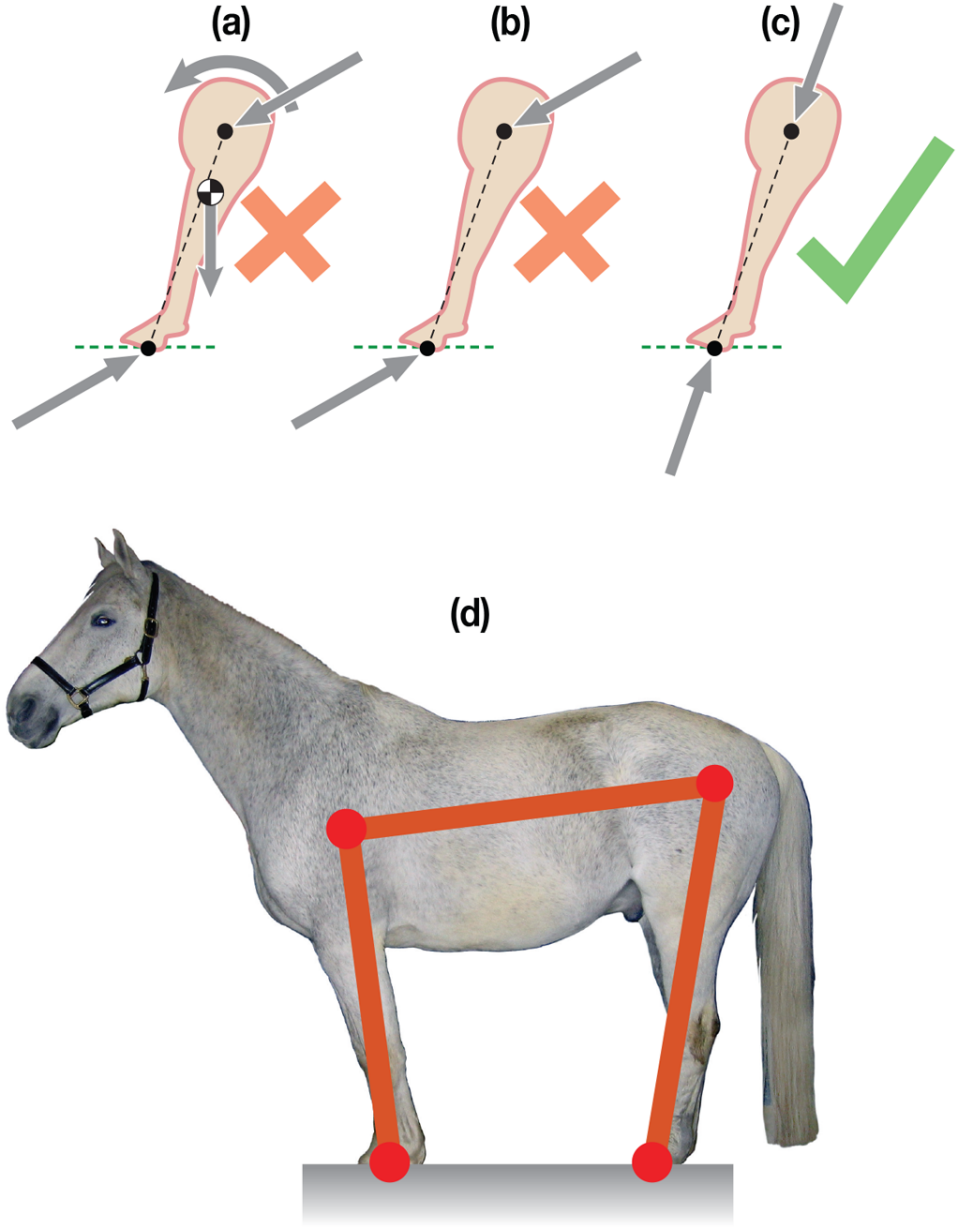
**Leg as a ‘two-force object’ and horse model.** Neglecting the weight of the legs, the legs are two-force objects. **(a) General case.** This free body diagram includes the leg weight and also torques from muscles at the hip. The laws of mechanics have not yet been applied. **(b) Two forces,** leaving off both the leg weight and also torques from muscles at the shoulder or hip. Force balance has been used but not yet moment balance, so the leg is not in equilibrium. **(c) The leg as a two-force body.** Leaving off the hip torque and the leg weight, force and moment balance necessitate that the two forces be equal and opposite and along the hoof-to-joint line. **(d) The horse model is made of three links:** the body, forelegs and rear (hind) legs; each leg pair is one link in the model. These three parts are connected with two hinges at the shoulder and hip.

For our 2D analysis, each leg pair is treated as a single object (e.g. we consider the fore legs as a single leg, Fig. 6**a–c**).

Forces and moments that are internal to the subsystem do not show on the free-body diagrams. For example, in the free-body diagram of the whole horse (Fig. 5), there are no muscle forces shown, because they act internally to that system (or, if you like, they act in action-reaction pairs that cancel).

#### A standing horse is a three-force object

First, consider the horse as a single stationary object with no concern for the presence, or absence, of muscular effort. We use unit vector 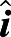 to indicate the direction to the right, towards the horse’s rear, and unit vector 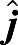 to indicate vertical upwards. There are three forces acting on the whole horse considered as a single object:

(1) The force of gravity, 

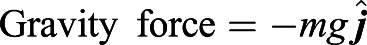
acting at the Center of Mass G of the horse;
(2) The total force of the ground on the front hooves (Fig. 6), 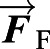, which can be decomposed in the vertical ***N***_F_ and frictional (horizontal) part ***H***_F_, with

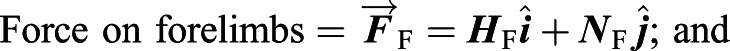
(3) The total force of the ground on the rear hooves, 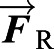, which can be decomposed in the vertical *N*_R_ and fictional (horizontal) part *H*_R_

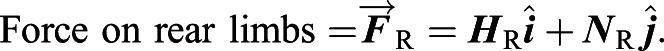

Neglecting the small motions during standing, and neglecting small forces (e.g. wind), we can apply the laws of statics, using only the two ground forces 
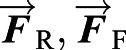
 and gravity 
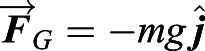
, three forces in total. Hence the horse is a so-called ‘three-force object’. The governing statics equations are:
(3)

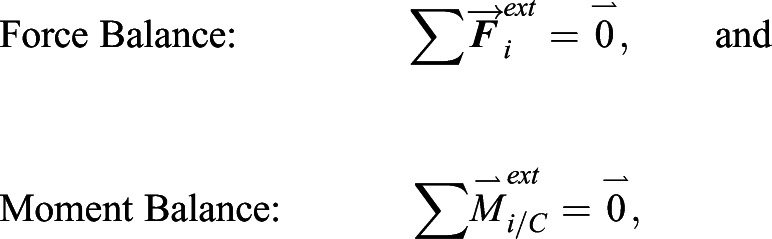
where the sums are over all of the external forces 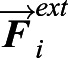 and moments 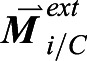 relative to C.

#### Features of three-force object equilibrium

As per any introductory statics book (e.g. Ruina and Pratap, 2019), we can use force and moment balance (Eqn. 3) to discover various features of the equilibrium forces.

1. **Three-force object.** Consider a point C at the intersection (if such exists) of the lines of action of the forces at the front hoof and at the rear hoof. The only force with moment about C is gravity. For there to be no net moment about C, the gravity moment must also be zero. So, moment balance about C implies that the two ground reaction forces, at the forelimbs and rear-limbs, are either (Fig. 5)
    i. vertical, or
   ii. have lines of action that intersect at a point directly C above G, or
  iii. have lines of action that intersect at C directly below G.
2. **The ground carries the weight.** The vertical component of force equilibrium implies that the vertical ground reaction forces add up to the weight: *N*_F_ + *N*_R_=*mg*
3. **Lever rule.** Moment balance about the point on the ground under the center of mass implies that *N*_F_*d*_F_=*N*_R_*d*_R_
4. **Vertical forces are determinate:** moment balance about the rear feet and about the front feet, together imply that the vertical ground reaction components are: *N*_F_=(*d*_R_/ℓ)*mg* and *N*_R_=(*d*_F_/ℓ)*mg*.
5. **Horizontal forces cancel but are indeterminate.** Force balance in the horizontal (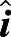) direction implies that the horizontal ground reaction forces are equal and opposite, *H*_F_=− *H*_R_. These forces are otherwise indeterminate (i.e. the magnitude of these canceling forces cannot be found from the laws of statics applied to a free-body diagram of the whole horse).

Any 2D statics model of a horse, and any measurement of a real stationary horse, must have forces obeying all of the above relations. These apply whether the leg weight is included, or not, and whether or not there are muscle contractions applying torque to the legs. These, above, are the strongest (i.e. most model-independent) results from statics as applied to whole horse in the sagittal plane.

### Approximating the legs as two-force objects

Before simplifying, we show a free-body diagram of the front leg pair or rear leg pair. Fig. 6a. This shows:

- including the ground reaction force 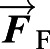 acting on the hoof at the ground. This ground reaction force 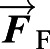 includes both a normal (vertical) component and a sideways frictional component;
- the gravity force on the leg *mg* pointing down;
- the joint force, from the body to the leg, at the shoulder 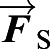; and
- *M*_S_, the moment of the muscles, tendons and ligaments at the shoulder. These act on the leg from the horse body.

We might assume that the horse chooses standing postures that minimize muscle tensions and thus minimize joint torques, so we assume zero net torque at the joints. Thus, the free body diagram of a leg (pair) has only two forces on it: one from the ground and one from the shoulder (or hip). So, we replace the free-body diagram in Fig. 6a with that in Fig. 6b. These forces can only be in equilibrium if the forces are equal and opposite and have a common line of action (‘two-force members’ or ‘two-force bodies’ in, e.g. Ruina and Pratap, 2019), as shown in Fig. 6c. Assuming no hip and shoulder motor torques, the forces on a horses leg, at the hip and ground, are well approximated as being equal and opposite and along the leg (along the line connecting the hip and hoof).

So, the ground reaction force at the front hoof points towards the shoulder and the ground reaction force at the rear hoof points towards the hip.

#### Equilibrium of standing: two-force legs and three-force horse

Because, assuming no muscle torques, the legs are two-force objects (Fig. 6) we can draw the free body diagrams shown in Fig. 5. That is, in addition to the hoof forces intersecting at a point above or below the center of mass, they are also along the lines from the hooves to the shoulder and hip.

For a pendulum we could recognize equilibrium by the center of mass being above or below the hinge. Whereas, for a horse the equilibrium posture, not using hip or shoulder muscles, has the lines along the legs intersecting directly above or below the horse center of mass. Or the lines along the legs are vertical.

The point of intersection C is partially analogous to the location of the hinge for a pendulum; for equilibrium the center of mass and point C (or the hinge) have to be vertically aligned. (In the special case of vertical hoof reaction forces one can think of the hinge as being at infinity, over or under the center of mass.)

Point C is also the instantaneous center of rotation of the horse body. Note, unlike the case for the hinge of a pendulum, which is fixed in space, we have found no significance, either for equilibrium nor for stability, in whether point C is above or below either G or the ground (note: the intersection of the two leg lines is not a fixed point of the moving mechanism and cannot be used for potential energy calculations).

### Calculation of stability

The stability calculations for the horse model are the same in spirit as the simpler calculations for an inverted pendulum (see Section *Pendulum analogy*).

#### One degree of freedom (DoF) mechanism

As described previously, our model of a standing horse is a ‘four-bar’ mechanism made up of three hinged objects (‘linked links’, Fig. 5). Given the position of the hooves and the lengths of the body parts, this linkage has one degree of freedom. That is, one number, say the angle *θ*_F_ of the fore-legs, determines all of the other positions and angles.

#### Dynamic stability

For any mechanical system near equilibrium, the governing equations are:
(4)

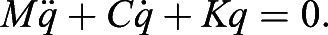
In this equation *q* is a list of numbers describing the deviation from equilibrium with 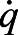 and 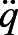 being the first and second time derivatives of *q*. The matrices *M*, *C* and *K* are constants (mass, damping and stiffness) that come from linearizing the equations of motion near the equilibrium. Assume that the mass matrix *M*, the damping matrix *C* and the stiffness matrix *K* are symmetric, the system is stable if the dissipation is always positive (i.e. *C* is positive definite) and if the associated potential energy is always positive (*K* is positive definite). That is, assuming positive dissipation, the system is dynamically stable if the equilibrium is at the bottom of a potential energy bowl.

In our case, with only one degree of freedom, *q* is just a single scalar number (say, the deviation of the hip joint angle from equilibrium), and *M*, *C* and *K* are also scalars. Assuming *C* is positive (damping is dissipative), stability is determined by whether *K*>0 (stable) or *K*<0 (unstable). That is, dynamic stability can be determined by calculations of potential energy in the neighborhood of the equilibrium (a statics calculation of sorts) while never doing a dynamics calculation. So, here we determine dynamic stability by looking at the net potential energy due to gravity (which contributes to making *K* negative) and due to the corrective springs (which contribute to making *K* positive).

#### Calculation of stability using potential energy

The calculation is done for a fixed value of the hoof spacing ℓ_*g*_ (analogous to a given ℓ/2−*d* on the pendulum). For that given ℓ_*g*_, at each configuration of the linkage there is a gravitational potential energy *E*_P_, which is the weight times the height of the center of gravity,
(5)

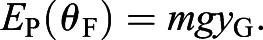
Note, to use this equation one must find the height *y*_G_ of the center of mass in terms of *θ*_F_ (unlike the pendulum, for the horse mechanism this is a non-trivial geometry/trigonometry problem with no simple formula). Any equilibrium posture (linkage configuration) is one in which all of the laws of statics hold for the whole horse and any of its parts. Assuming legs with negligible mass and with negligible muscle and ligament torques, the possible equilibrium configurations are shown in Fig. 5. A theorem from statics is that an equilibrium configuration is also one for which the potential energy is ‘stationary’, meaning,
(6)

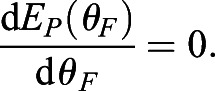
This means that the equilibrium (zero muscle force) posture of a horse is at a (local) potential energy maximum, or minimum (or, a ‘stationary’ point). In all of our calculations for semi-realistic horse proportions, we find the equilibrium postures to be at a local maximum of potential energy; the energy function is an upside-down bowl. That is, we always find that when we find a *θ*_F_ that satisfies Eqn. 6 that it also satisfies,
(7)

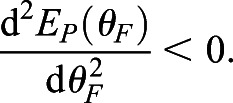
Such an equilibrium, at a potential energy maximum, is unstable. If disturbed ever-so-slightly from this configuration the horse would, if there were no ligaments, muscle forces or other joint torques, fall down or at least fall to a cockeyed equilibrium shape. That is, in our three-link model, a standing horse in equilibrium is, with no muscle effort, unstable. Nonetheless, horses do stand for extended time without falling.

To do this, horses must apply corrective torques. We assume mostly tonic mechanisms for this correction with the feature that deviations from equilibrium posture lead to proportional corrective torques. Thus, tonic muscle contractions are much like torsional springs, *k*_F_ and *k*_H_, at the joints. Corrective joint torques when the joint angles, *θ*_F_ and *θ*_H_, deviate from equilibrium, are:
(8)

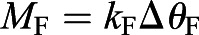
and
(9)

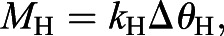
where Δ*θ*_F_ and Δ*θ*_H_ are the deviations of the joint angles relative to equilibrium. The addition of these springs does not change the equilibrium posture, but the springs do change the stability. Namely, the potential energy is now:
(10)

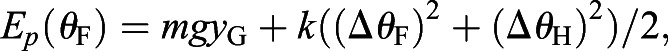
where for simplicity of modeling we use the same spring constant at both joints, i.e. *k*=*k*_F_=*k*_H_. For a given horse model with given hoof placement we can solve for the equilibrium posture (configuration). And using Eqn. 10 we can also find the minimum value *k*_min_ of *k* in order to make the posture stable. That is, we apply Eqn. 10 to the critical condition (inequality in Eqn. 7 used at equality),
(11)

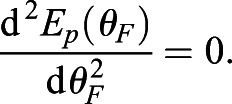
and solve for *k*, calling that *k*_min_.

For each hoof spacing, canted-in, vertical, or splayed-out, we can find the associated equilibrium posture one of two ways: either using the two-force-object and three-force-object reasoning above (see Section *Finding horse equilibrium postures*), or by finding stationary points for the gravitational potential energy. As noted earlier, the model allows some non-physical postures, for example, hanging upside down from the ground surface. Depending on leg and body lengths, there can also be very-crooked equilibrium postures, with the hip much above or much below the shoulder. And postures above and below ground with the legs crossed and the body possibly upside down. We exclude all of these. There is just one above-ground posture that has the legs not crossed, the body near level and that satisfies the equilibrium equations. For this one unstable equilibrium above-ground posture, we seek the minimum needed stiffness *k*_min_ at the hip and shoulder for it to gain stability. Because of the complex geometry of the linkage, and lack of symmetry, both the find-equilibrium and evaluate-stability calculations are done numerically.

### Numerical methods

We parameterize the first postural choice, leg splay, by the hoof-to-hoof ground spacing, ℓ*_g_*.

#### Find equilibrium geometry using numerical root finding

Given ℓ*_g_* we then consider a candidate value for the slope *θ*_ F_ of the fore-legs. Given *θ*_ F_, the law of sines and the law of cosines finds the full 4-bar linkage geometry, including all link angles and all joint positions. We then used numerical root finding to find that value of *θ*_ F_, which put the intersection of the two leg lines directly above, or directly below, the horse center of mass. That configuration is, as per the three-force-object reasoning, the equilibrium standing configuration for the given leg splay. Alternatively, we could have done numerical optimization (maximization) of the potential energy.

#### Numerical calculation of stability

Given the equilibrium configuration for given ℓ*_g_*, we then found the equilibrium configuration's stability as follows. We varied *θ*_ F_ in the neighborhood of the equilibrium position. For each *θ*_ F_ we found a consistent linkage geometry and associated center-of-mass height and hence (multiplying by *mg*) potential energy. We then did a polynomial fit of that function and recorded the quadratic term. We similarly found the best fit for the sums of squares of the shoulder and hip joints versus *θ*_ F_ in the neighborhood of equilibrium. Given those two fits, we could find that torsional spring constant *k* so that if one such spring was put at the shoulder, and another at the hip, that the net quadratic term in the potential energy would be zero. This is the so-called ‘minimum required joint stiffness’ *k*_min_, displayed graphically in Fig. 3.

### Determining model parameters

The horse (Fig. 5) is made of three rigid objects: (1) the fore-legs with length ℓ_ F_; (2) the rear legs with length ℓ_R_; and (3) a back with length ℓ_*b*_ connecting the shoulder S to the hip H. The hoof ‘points’ F and R are at the centers of pressure of the hoof ground reaction forces (i.e. the points where the equivalent single ground-reaction force-couple systems have no net torque). We do not attempt to find this point accurately and take it to be at the rough ‘middle’ of the footprint. The center of mass G is a distance *d*_G_ back from S along SH, and *h*_G_ orthogonally above the line SH. The distance between the legs, from F to R, is ℓ*_g_*=*d*_ F_+*d*_R_, where *d*_ F_ and *d*_R_ are the distance from the ground-projection of G to the front and rear hooves, respectively.

We created a ‘standard horse’ based on measurements from a few real horses. We scaled pictures to a common size so that pictures could be overlaid and compared. We scaled to a 16-hand horse, 64″ from ground to withers (the high part of the back, just behind the neck).

#### Location of shoulder and hip joints

**Fig. 7.**
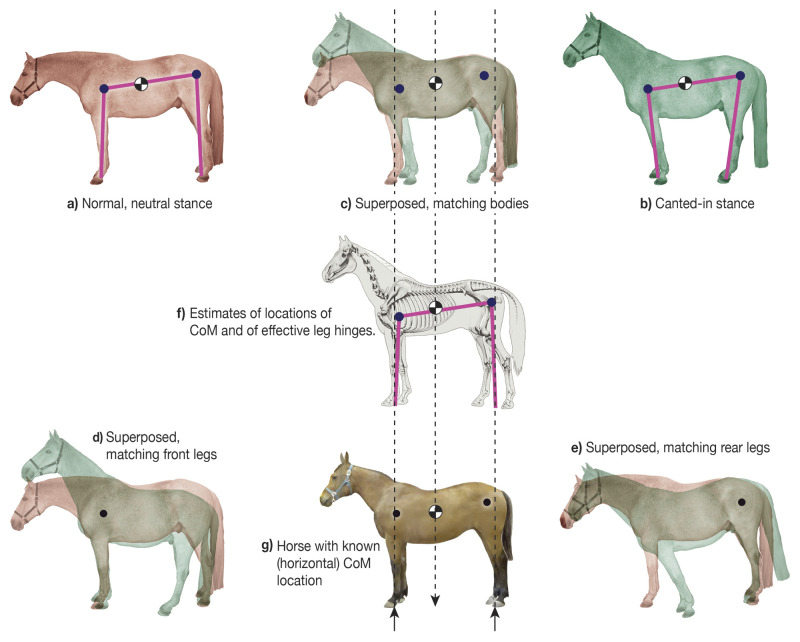
**Locating the CoM and the effective shoulder and hip.** One horse was photographed twice, **(a)** once in normal posture and **(b)** once with legs canted-in, and **(c)** shown superposed. By rotating the photos **(d,e)**, locations are found for which relative rotation about those points best fits the observed motion of the leg relative to the body **(d,e)** and the body relative to the leg **(c)**. **(g)** With a different horse, we found the CoM using four force plates (Gellman, Shoemaker, and Reese, unpublished data). All three horse images are re-scaled to about the same size so that the effective hinges and the CoM can be shown on all. For calculations, the dimensions for the effective leg lengths, back length, and CoM location on the back, are measured from the photos, assuming a 16 hand (1 hand=4″) horse. Photos by J.M. Shoemaker.

Location of the effective shoulder joint and hip joint was done by superimposing photos of a single horse with more and less splayed legs (Fig. 7). The effective shoulder is that point about which we can rotate one picture relative to the other so that in one case the legs are perfectly aligned and in the other the bodies are perfectly aligned. This point was found bysequential guessing using a graphics program (Adobe Illustrator). The hip was found similarly. All distances were measured from the scaled pictures.

#### Parameter values

*m*=: 500 kg. More or less typical for a 16-hand horse;
*g*=: 10 m/s^2^. Close enough to the standard value of 9.8 m/s^2^;
ℓ_F_=: 1.05 m. Fore-leg length, ground to shoulder hinge;
ℓ_R_=: 1.20 m. Rear leg length, ground to hip hinge;
ℓ_***b***_=: 1.16 m. Body length, from shoulder hinge to hip hinge;
*d*_G_=: 0.50 m. Distance along the shoulder to hip line from the shoulder hinge to the CoM.
*h*_G_=: 0.0 m. We assume the CoM was on the shoulder to hip line, thus the height of the CoM above that line is zero.

### Modeling choices A: basic features

Here is an expanded discussion of the modeling choices given at the start of the Materials and Methods section.

#### 2D versus 3D model

We use a 2D analysis here. We look at the horse from the side, considering the *sagittal=side-view=lateral-view* plane. We use a 2D, instead of 3D, analysis because the 2D results are simpler to understand than 3D results. In particular, the fewer the number of forces and force components to consider, the more accessible are the results. Further, the 2D results are exact features of the 3D mechanics. That is, if all 3D forces are projected into the sagittal (side view) plane, and the forces on all horse parts are considered as the set on the left and right pair added (e.g. the rear legs ground force is the sum of all forces on both rear feet), then the 2D statics results are exact results for the projections of the 3D forces and moments. The ‘four-bar’ linkage model here for a horse's sagittal plane is the same linkage used to model to the frontal plane of standing cats (forelimbs only) and humans in (Scrivens et al., 2006; Bingham et al., 2011) and (Goodworth et al., 2014). Like Scrivens et al. (2006) and Bingham et al. (2011), our model has no other moving parts [in contrast, Goodworth et al. (2014) adds an independently moving upper body].

#### The horse as a quasi-static mechanism

As noted previously, we use the common approximation that an animal is a linkage of rigid parts connected by hinges. During quiet stance, the lower leg-joints of the weight-bearing legs are generally locked in an extended (straight-legged) configuration; horses have special purpose anatomical features (the stay apparatus) that keep the leg straight with minimal muscular effort. Also, there is little deformation of the back. So, we assume that both stabilizing the spine, and holding the legs straight, are either non-problematic for a horse, or are otherwise solved problems. Of interest here are only the rotations of the legs, relative to the back, at the shoulder and hip joints. By ‘shoulder’ and ‘hip’ we mean the effective centers of rotation of the legs relative to the body (as found above). Our simplification of dynamics to statics is in contrast to the more complete dynamics postural models of Scrivens et al. (2006), Bingham et al. (2011) and Goodworth et al. (2014). Because the legs are much lighter than the body, we neglect the leg weights. Thus, we consider the statics of a 2D horse consisting of a rigid body (trunk, neck and head), with mass, which is supported by negligible-mass, rigid fore-legs and rigid hind-legs.

#### The linkage model and what it neglects

In a linkage model, each link represents a bone and the flesh on that bone. Deformations of both bone and flesh are neglected.

The torques (moments) transmitted across a real horse's joints can be from various sources:

1. Joint friction, which is presumed negligible for our simple analysis;
2. complex joint contact wherein there is not a single (e.g. center-of-sphere) effective hinge location, instead we assume simple pin joints;
3. Tension in muscles and tendons. These are of central concern for the stability calculations here but are neglected in the equilibrium calculations;
4. Tension in ligaments, which we consider as part of the mechanism constraints (e.g. enforcing the approximate pin-like joint kinematics). We assume that these have negligible torque about the nominal joint axes; and
5. Other soft tissue stresses (e.g. skin tension), which we assume are negligible.

#### Base of support versus mechanism instability

Note, we are not concerned with the horse falling over in the way that a rigid toy topples; the simple idea that a broader stance is more stable because the base of support (the polygon defined by the hoof positions) is bigger, is *not* key as noted in Goodworth et al. (2014). Rather, in a muscle-free linkage model, a standing horse is an unstable mechanism. This mechanism is prone to undesirable changes of internal degrees of freedom of the linkage, with, all the while, all feet firmly on the ground. This linkage instability, which is our concern here, must be controlled even when there is no danger of the center of mass falling outside the base of support.

#### The stance-angle effect on leg compression force is small

A first thought is that vertical legs would be preferred to canted-in or splayed-out legs because the compression in the leg is minimized with vertical legs. For the consideration of equilibrium forces, we lose little accuracy by replacing our model with one in which the fore and rear legs have equal length. The compression in the legs, *F*_c_, is then proportional to *W*/cos *θ*, where *W* is the weight born by the leg, if vertical and *θ* is the deviation from vertical. For small angles, this is approximately quadratic in leg angle, *F*_c_≈*W*(1+*θ*^2^/2) (where *θ* is measured in radians) and is not a dramatic effect. For example, for 
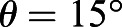
 the compression is increased by 3.5% compared to having vertical legs (
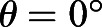
). Canted-in or splayed-out limb angles seen in living horses are usually well under 
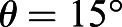
 (Gellman, Shoemaker and Reese, unpublished data). That is, while leg compression force is minimized by having vertical legs, it is a broad minimum and thus likely has small effect on postural choice, at least within the range of angles (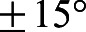 that we consider here.

In short, the leg compression varies only quadratically with small deviations from vertical whereas the needed stiffness for stabilization *k*_min_ varies linearly. The linear (larger) effect likely dominates.

## Modeling choices B: Tonic stiffness; corrective feedback torques; and the effects of feedback delays

Our concern is the stabilization of a skeletal configuration that is in unstable equilibrium. In our model, this is a standing horse. Others have modeled humans standing in the sagittal (side view) or frontal plane (e.g. Loram et al., 2005a,b; Scrivens et al., 2006; Bingham et al., 2011; Li et al., 2012; Goodworth et al., 2014). A related ‘posture’ problem is human grasp using fingertips (e.g. Sharma and Venkadesan, 2022), in which the relevant skeletal configuration would be the finger geometry. Another ‘posture’ problem could be an arm pushing a door (Rancourt and Hogan, 2001); posture would be the configuration of the upper-body, hand and arm. In the grasping and pushing tasks, the equilibrium involves torques from muscles. Even accounting for the intrinsic stiffness of the torque-generating muscles, candidate equilibrium configurations of a finger pinch or arm push might be unstable. In contrast with pinching or pushing, which necessarily use muscles, equilibrium standing does not need muscles (until we consider stability). Whether the load causing the potential instability is generated by muscles (as in finger pinching or door pushing), or is external, say from gravity (e.g. standing), the animal or human may need to supply additional stabilizing torques to hold the desired equilibrium posture (that is, to avoid buckling-like collapse).

### Types of corrective torques

In all plausible postures, we found that without corrective torques, a horse's standing posture is unstable. There are some possible non-muscular ways a horse could apply these torques (see Section *Modeling choices A*). This paper focuses on the corrective torques from muscles (in series with tendons).

The corrective torques are of two general kinds: (1) tonic stiffness; and (2) feedback correction. The tonic stiffness can, in turn, come from (a) co-contraction; (b) the intrinsic stiffness of the muscles used in the task, and (c) the intrinsic stiffness of muscles used to hold a posture slightly ‘leaned’ away from equilibrium. These are discussed in more detail below.

**1. Tonic stiffness** is corrective torque from muscle's intrinsic stiffness. This is the slope of the muscle's force-length relation in a quick (too fast for neural feedback) imposed extension (neglecting, or subtracting out, viscous or hysteretic effects). As pointed out in De Groote et al. (2017), this ‘short-range’ (i.e. intrinsic, short time) intrinsic stiffness does not involve feedback via neural control. As is well known and noted in, e.g. Sharma and Venkadesan (2022), this intrinsic muscle stiffness is more-or-less proportional to the muscle force present. So, intrinsic stiffness is only significant if a muscle is activated. We term the stabilization from intrinsic stiffness due to constant muscle activation as ‘tonic’. It is also ‘passive’ in that it does not involve active neural control. It is also a primitive form of ‘feed-*forward* control’ in that it is a chosen activation pattern, albeit simple, that achieves the desired coordination without feed*back*. There are three mechanisms for tonic stiffness in a static posture:
  a. **Co-contraction,** with flexors and extensors in opposition;
  b. **Tonic stiffness** from the task. For human grasp, the propensity to buckle (negative stiffness) fights the intrinsic muscle stiffness. Because force (destabilizing) and intrinsic stiffness (stabilizing) are nearly proportional, the stability, or lack thereof, of a given grasp configuration is approximately unchanged by the tightness of the grasp (Sharma and Venkadesan, 2022). In our horse standing model, we assume that the horse chooses an equilibrium configuration that requires no hip or shoulder torques, so this mechanism (stiffness from muscles already needed for a task) is not relevant.
  c. **Contraction opposing an external load.** For example, this is thought to be an approximation of the state of a typical standing person [e.g. Loram and Lakie (2002) and see Figure 1 in Loram et al. (2005b)]; the person is leaned forward in the sagittal plane, relative to muscle-free equilibrium, with their center of mass slightly forward of the ankle. The external gravity load causes a disruptive torque (about the ankle hinge) and the Achilles tendon (with soleus and/or gastrocnemius muscles) provides a counter-acting righting torque.
    In a standing person or horse, as the deviation from muscle-free equilibrium is increased, the bigger the muscle force needed to balance the gravity force. Meanwhile, the negative stiffness from gravity is essentially independent of the magnitude of deviation. Therefore, the bigger the chosen distance from muscle-free equilibrium, the bigger the intrinsic muscle stiffness, the bigger the ratio of muscle stiffness to gravity negative stiffness, the more stable is the system. The horse can thus choose a stiffness by choosing an amount of deviation from equilibrium, something like the choice a human can make stabilizing a ladder. Again, consider a tall, heavy, vertical ladder. You want to stabilize it without using feedback or co-contraction. The ladder will need to lean sufficiently so that the force to stop it from falling is high enough to engage stiff-enough muscles to stabilize the position. This useful increase in muscle stiffness with load (and distance from equilibrium) is in contrast with grasp. In grasp, as per (1b) above, the increase in load does not help make an unstable system stable.
    {Note, for our horse calculations we calculate the needed stiffness in a muscle-free equilibrium configuration. For the mechanism of generating tonic stiffness without muscle co-contraction, this stiffness depends on leaning a small amount away from the muscle-free equilibrium [Figure 1 in Loram et al. (2005b)]. We assume that the geometry change from that leaning is small-enough as to have negligible effect on the needed value of *k*_min_]}.

**2. Feedback correction torques** in response to sensed deviations from equilibrium. These senses could be on proprioceptive (mechano-receptors distributed in muscles, joints, ligaments and skin), visual (oculo-vestibular responses), or inner ear (vestibular end organs – otoliths and semicircular canals). These signals are processed in the spine or brain, and then lead to muscle commands. This corrective strategy might be termed as ‘feedback’, in that activation based on sensing is fed back to the muscles, and as ‘active’, in that the neural output is actively changing.
  Active neural feedback in an animal (or human) is not instantaneous due to sensor delay, neural transit times to the spine or brain, neural processing time, neural transit time back to the muscle, and muscle delays (so called ‘activation delays’, due to electrical, chemical and mechanical muscle processes). These delays have a variety of delay times, depending on the pathways. Added up along a single feedback pathway these are thought (for humans) to be in the range of 0.1–0.3 s (Goodworth et al. 2014).

**Combining passive tonic and active feedback controls.** For human standing balance in the sagittal (side view) plane, Loram and Lakie (2002) show that there is some stiffening from the intrinsic stiffness of active calf muscles fighting gravity (tonic mechanism 1c above). On top of that there is a dynamic feedback loop. Similarly, tonic stiffness from co-contraction (1a above) can be supplemented with active feedback,
  “[For stable upright balance, to counteract the negative stiffness from gravity] the required torsional stiffness may be provided by negative position feedback or by antagonist coactivation, or a combination of both” (Hogan, 1984).

It is sensible that an animal or person would choose to stabilize using a mixture of tonic (constantly contracted muscle) and feedback control (sense and response), for three reasons:

1. There is a cost to varying muscle force. This cost is on top of the cost of maintaining that force. Given that the sensors have limited resolution, feedback control necessarily involves force variation. If some of the stability is handled by tonic contraction (using intrinsic muscle stiffness), then there is less instability for the feedback system to contend with, and, for given sensor thresholds, smaller needed corrective torques. Depending on details of muscle costs for tonic forces versus the costs for the necessarily time-varying contractions used in feedback, there could be a savings of chemical energy by using a mixture of tonic (intrinsic stiffness) and feedback control (Art Kuo, personal communication);
2. As noted previously, muscles are less stiff when the force is smaller. Starting with no force, to generate a small force, slack needs to be taken up. Whereas, when a muscle is already contracted, it takes less change in excitation to cause a similar force change, and there is less related muscle and tendon stretch. That is, modulating an existent force may be easier than modulating a force near to zero force (Art Kuo, personal communication); and
3. The system may be so unstable that, with the given neuro-muscular delays and given sensor inaccuracy, that it is uncontrollable using feedback alone, and tonic stabilization is needed.
  This last case is apparently observed in Sharma and Venkadesan (2022) for pinched grasp. For that task, with just enough muscle tension to accomplish a given pinch force, the posture is unstable. These instabilities are associated with a characteristic time *τ*_inst_ [meaning that a variable-of-interest *x* diverges from a desired configuration exponentially in time as *x*(*t*)∼exp (*t*/*τ*_inst_)]. In principle, with perfect sensing, accurate actuation, and no external disturbances, these instabilities can be stabilized with feedback that uses vanishingly small muscle effort, even with time delay *τ*_delay_,
  In contrast, in practice there are imperfections (sensing and modeling errors ε_sense_, actuation errors ε_act_, disturbances *f*_dist_), and some muscle effort meffort is required for stabilization. Assume one needs a given accuracy of control of *x*<*x*_max_, where *x*_max_ corresponds to failure, such as falling down. The requirement for accuracy (in sensors and actuators) and need for disturbances on the system to be small, grows exponentially with time delay — the longer the delay, the greater accuracy is needed. That is, roughly,
  Hence, the practical not-quite-rule that you cannot control an unstable system if the delay time *τ*_delay_ is longer than the characteristic instability time *τ*_inst_. It is possible to control with longer delays, but the demands for system precision grow exponentially with the amount of delay. Furthermore, the needed actuator (muscle) effort also grows.
  For a given system, the delays and system imperfections are what they are. And the demand for accuracy for a given task is what it is. If the delay time *τ*_delay_ is significantly larger than *τ*_inst_ then achieving *x*<*x*_max_ might not be possible, as seems to be the case for finger grasps (Sharma and Venkadesan, 2011).

Taking the three mechanisms above into account, we might consider the general motor-control principle that passive mechanisms, (tonic muscle stiffness, or postural choices, or even skeletal design), may be used to bring a system to a point close to neutral stability, and that feedback is then used to do the remaining control of the resulting almost-stable (meaning slow instability times) system.

### Tonic mechanism as rationale for *k*_min_ measure of instability

The key idea justifying our standing horse model here is that a horse might well use tonic mechanisms to bring their intrinsically unstable posture up to, or almost up to, neutral stability. If this is the case, our quantification of the difficulty of stabilizing a posture using *k*_min_ seems reasonable; *k*_min_ represents the tonic contraction, and thus effort, needed to just-stabilize a posture.

### Comparison with Bingham et al. (2011)

In the study of the effect of leg spread on the stability of human standing, (ibid.) ignore the intrinsic stiffness of muscles, only considering active feedback mechanisms. Central to their model and calculations is that this feedback has neuromuscular delays.

Their analysis of the stability of humans in the frontal (side to side) plane uses, like we do here, a four-bar linkage model. Their mass, length (and inertia) parameters model those for a human, our parameters (with no consideration of inertia) are for a standing horse. Their two key model findings are:

1. They notice that their model is *less* stable with spread legs than with vertical legs, and
2. They find that there is a range of feedback gains which stabilize the system. This range is smaller for spread legs than for parallel, vertical legs.

These results are, at least superficially, in contrast with our model results that:

1. Our horse is *more* stable with legs splayed-out than with legs vertical (parallel), and
2. The minimum stiffness needed for stabilization is less for a splayed-out horse than for a vertical-legs horse.

Without checking the models in detail, we believe this superficial contradiction is not due to the difference in size and shape between horses and humans; they would have got their same results (a wider stance is harder to stabilize) if they had modeled horses and we would have got our same results (that a wider stance is easier to stabilize) if we had modeled humans.

Besides the differences in physical parameters (lengths, weights), which we do not think are key to our different results, our model differs from (Bingham et al., 2011) in two key ways:

A. They use the eigenvalue *λ* as a measure of the degree of instability whereas we use the minimum corrective spring *k*_min_, and
B. They assume all control is with neural feedback that has a delay, whereas, we do not directly consider delay.

One Bingham observation might be explained intuitively as follows: the splayed-out posture is associated with a smaller inertia (less kinetic energy per unit leg angular rate). Thus, although the gravitational negative stiffness is smaller with splayed-out legs, the even-more reduced inertia makes for a reduced characteristic time of falling (bigger eigen value). By their speed-of-instability measure, a splayed-out posture is thus less stable. On the other hand, when the legs are splayed-out, a given motion of the center of mass is associated with large changes in the body-to-leg angles, so that our model's joint springs have a larger stabilization effect. Thus, by our *k*_min_ measure, splayed-out is more stable.

Bingham et al.’s main result is that splayed-out is, by their measure, harder to control. Consistent with us, they find that the minimum gains to stabilize the splayed-out posture are smaller. But their model includes delay. If one uses a gain much larger than the minimum-needed gain, then, in their dynamics model, the characteristic sway becomes faster with shorter characteristic times, eventually competing with neuromuscular delay times, and the control system becomes unstable (there is an oscillatory instability at a critical gain).

On the other hand, at narrower stance, a higher control gain is needed (in both Bingham et al. and our model). And, again, at sufficiently higher gain, their model becomes unstable. Their central observation is that *they find a smaller range of gains is stabilizing for wide stance than for narrow stance.*

Thus, they reason, a control system that is challenged in its abilities to set gain levels, has an easier time setting the gain in narrow stance.

In summary, relative to a narrow stance, in a wider stance the needed feedback control gains are smaller (whether with or without delay), the characteristic time of instability is smaller, the needed stabilizing spring *k*_min_ is smaller, and, if delays are included, the range of stabilizing gains is smaller. While they did not calculate horse geometries and we did not calculate human geometries, this collection of things seems to be true for both horses in side view and people in front view. If tonic mechanisms are used to make the person/animal stable, or nearly so, the Bingham et al. model seems inappropriate. If tonic mechanisms are not used at all, our model seems inappropriate.

### Lack of ‘objectivity’ in Bingham et al. (ibid.)

Bingham et al.'s instability measure is not ‘objective’ in that the result depends on the choice of measure. We do not know how a biological neural system quantifies/measures gains. The concept of ‘narrower gain range’ depends on how gain is measured. For example, the neural system might use the log of the engineering gain, or the reciprocal (the compliance). A range of gains that is smaller using engineering gain may not be smaller using one of these, or some other measure. That is, the concept of ‘smaller gain range’ is not *objective*; the phrase ‘smaller range of gains’ is not independent of the means of measuring. If one range of gains totally encompasses another range of gains, then one range being bigger than another is objective (is independent of what measure of gain is used). But one stiffness range does not encompass the other in their model. In summary, the phrase ‘smaller stable range’ depends on how gain is parameterized (e.g. engineering gain versus reciprocal of engineering gain, versus log of engineering gain, etc.), so their smaller range may or may not be smaller with a biologically appropriate measure of gain.

### Parkinson's disease versus other challenges

The Bingham et al. model is primarily intended to explain the narrow stance of people with PD. In most (non-Parkinsonian) situations where balance is a challenge (neural ataxia), it seems that more-spread stances are chosen by both humans (wider) and horses (longer) (Alcott, 2017; Bunn et al., 2013), not the narrower stance observed for standing human PD patients. Bingham et al. (2011) do not claim that their model is predictive in these other situations.

## References

[BIO059139C1] Alcott, C. (2017). Evaluation of ataxia in the horse. *Equine Vet. Educ.* 29, 629-636. 10.1111/eve.12461

[BIO059139C2] Baxter, G. M., Stashak, T. S. and Keegan, K. G. (2020). Chapter 2. In *Examination for Lameness*, p. 85. John Wiley & Sons, Ltd.

[BIO059139C3] Bingham, J. T., Choi, J. T. and Ting, L. H. (2011). Stability in a frontal plane model of balance requires coupled changes to postural configuration and neural feedback control. *J. Neurophysiol.* 106, 437-448. 10.1152/jn.00010.2011 21543754 PMC3129728

[BIO059139C4] Bunderson, N. E., Burkholder, T. J. and Ting, L. H. (2008). Reduction of neuromuscular redundancy for postural force generation using an intrinsic stability criterion. *J. Biomech.* 41, 1537-1544. 10.1016/j.jbiomech.2008.02.004 18374342 PMC4121430

[BIO059139C5] Bunn, L. M., Marsden, J. F., Giunti, P. and Day, B. L. (2013). Stance instability in spinocerebellar ataxia type 6. *Mov. Disord.* 28, 510-516. 10.1002/mds.25163 23143967

[BIO059139C6] Clayton, H. M., Bialski, D. E., Lanovaz, J. L. and Mullineaux, D. R. (2003). Assessment of the reliability of a technique to measure postural sway in horses. *Am. J. Vet. Res.* 64, 1354-1359. 10.2460/ajvr.2003.64.1354 14620769

[BIO059139C7] Clayton, H. M., Buchholz, R. and Nauwelaerts, S. (2013). Relationship between morphological and stabilographic variables in standing horses. *Vet J.* 198, e65-e69. 10.1016/j.tvjl.2013.09.035 24144772

[BIO059139C8] De Groote, F., Allen, J. L. and Ting, L. H. (2017). Contribution of muscle short-range stiffness to initial changes in joint kinetics and kinematics during perturbations to standing balance: A simulation study. *J. Biomech.* 55, 71-77. 10.1016/j.jbiomech.2017.02.008 28259465 PMC5436583

[BIO059139C9] Divers, T. J., Mohammed, H. O. and Cummings, J. F. (1997). Equine motor neuron disease. *Vet. Clin. North Am*. 13, 97-105. 10.1016/S0749-0739(17)30258-4 9106346

[BIO059139C10] Fraser, J. E. (1918). *End of the Trail*. https://www.metmuseum.org/exhibitions/listings/2013/the-american-west-in-bronze/blog/posts/end-of-the-trail.

[BIO059139C11] Fureix, C., Hausberger, M., Seneque, E., Morisset, S., Baylac, M., Cornette, R., Biquand, V. and Deleporte, P. (2011). Geometric morphometrics as a tool for improving the comparative study of behavioural postures. *Naturwissenschaften* 98, 583-592. 10.1007/s00114-011-0803-2 21573691

[BIO059139C12] Garcia, M., Chatterjee, A. and Ruina, A. (2000). Efficiency, speed, and scaling of two-dimensional passive-dynamic walking. *Dyn. Stab. Syst.* 15, 75-99. 10.1080/713603737

[BIO059139C13] Gatesy, S. and Biewener, A. (1991). Bipedal locomotion: effects of speed, size and limb posture in birds and humans. *J. Zool.* 224, 127-147. 10.1111/j.1469-7998.1991.tb04794.x

[BIO059139C15] Goodworth, A. D., Mellodge, P. and Peterka, R. J. (2014). Stance width changes how sensory feedback is used for multisegmental balance control. *J. Neurophysiol.* 112, 525-542. 10.1152/jn.00490.2013 24760788 PMC4122700

[BIO059139C16] Heglund, N., Fedak, M., Taylor, C. and Cavagna, G. (1982). Energetics and mechanics of terrestrial locomotion. iv. total mechanical energy changes as a function of speed and body size in birds and mammals. *J. Exp. Biol.* 97, 57-66. 10.1242/jeb.97.1.57 7086351

[BIO059139C17] Hogan, N. (1984). Adaptive control of mechanical impedance by coactivation of antagonist muscles. *IEEE Trans. Autom. Control* 29, 681-690. 10.1109/TAC.1984.1103644

[BIO059139C18] Hoyt, D. F., Wickler, S. J., Dutto, D. J., Catterfeld, G. E. and Johnsen, D. (2006). What are the relations between mechanics, gait parameters, and energetics in terrestrial locomotion? *J. Exp. Zool. A Comp. Exp. Biol.* 305, 912-922. 10.1002/jez.a.335 17029281

[BIO059139C19] Kim, S., Horak, F. B., Carlson-Kuhta, P. and Park, S. (2009). Postural feedback scaling deficits in parkinson's disease. *J. Neurophysiol.* 102, 2910-2920. 10.1152/jn.00206.2009 19741108 PMC2777824

[BIO059139C20] Kingma, H., Gauchard, G. C., De Waele, C., Van Nechel, C., Bisdorff, A., Yelnik, A., Magnusson, M. and Perrin, P. P. (2011). Stocktaking on the development of posturography for clinical use. *J. Vestib Res.* 21, 117-125. 10.3233/VES-2011-0397 21558637

[BIO059139C21] Kram, R. and Taylor, C. R. (1990). Energetics of running: a new perspective. *Nature* 346, 265-267. 10.1038/346265a0 2374590

[BIO059139C22] Lesimple, C., Fureix, C., De Margerie, E., Sénèque, E., Menguy, H. and Hausberger, M. (2012). Towards a postural indicator of back pain in horses (equus caballus). *PLoS One* 7, e44604. 10.1371/journal.pone.0044604 22970261 PMC3436792

[BIO059139C23] Li, Y., Levine, W. S. and Loeb, G. E. (2012). A two-joint human posture control model with realistic neural delays. *IEEE Trans. Neural Syst. Rehabil. Eng.* 20, 738-748. 10.1109/TNSRE.2012.2199333 22692939

[BIO059139C24] Loram, I. D. and Lakie, M. (2002). Direct measurement of human ankle stiffness during quiet standing: the intrinsic mechanical stiffness is insufficient for stability. *J. Physiol.* 545, 1041-1053. 10.1113/jphysiol.2002.025049 12482906 PMC2290720

[BIO059139C25] Loram, I. D., Maganaris, C. N. and Lakie, M. (2005a). Active, non-spring-like muscle movements in human postural sway: how might paradoxical changes in muscle length be produced? *J. Physiol.* 564, 281-293. 10.1113/jphysiol.2004.073437 15661825 PMC1456051

[BIO059139C26] Loram, I. D., Maganaris, C. N. and Lakie, M. (2005b). Human postural sway results from frequent, ballistic bias impulses by soleus and gastrocnemius. *J. Physiol.* 564, 295-311. 10.1113/jphysiol.2004.076307 15661824 PMC1456055

[BIO059139C27] Mayhew, I., Jolly, R., Burnham, D., Ridler, A., Poff, G. and Blair, H. (2013). Familial episodic ataxia in lambs. *N Z Vet. J.* 61, 107-110. 10.1080/00480169.2012.717501 22985028

[BIO059139C28] McGeer, T. (1990). Passive dynamic walking. *Int. J. Robotics Res.* 9, 62-82. 10.1177/027836499000900206

[BIO059139C29] Meijaard, J., Papadopoulos, J. M., Ruina, A. and Schwab, A. (2011). History of thoughts about bicycle self-stability. *ecommons.cornell.edu*. https://ecommons.cornell.edu/handle/1813/22497

[BIO059139C30] Morasso, P., Casadio, M., Mohan, V., Rea, F. and Zenzeri, J. (2015). Revisiting the body-schema concept in the context of whole-body postural-focal dynamics. *Frontiers in Human Neuroscience* 9, 83. 10.3389/fnhum.2015.00083 25741274 PMC4330890

[BIO059139C31] Rancourt, D. and Hogan, N. (2001). Stability in force-production tasks. *J. Mot. Behav.* 33, 193-204. 10.1080/00222890109603150 11404214

[BIO059139C32] Ruina, A. and Pratap, R. (2019). *Introduction to Statics and Dynamics*. Self-published.

[BIO059139C33] Scrivens, J. E., Ting, L. H. and DeWeerth, S. P. (2006). Effects of stance width on control gain in standing balance. In 2006 International Conference of the IEEE Engineering in Medicine and Biology Society. 10.1109/IEMBS.2006.259876 17947066

[BIO059139C34] Shakeshaft, A. and Tabor, G. (2020). The effect of a physiotherapy intervention on thoracolumbar posture in horses. *Animals* 10, 1977. 10.3390/ani10111977 PMC7693906 33126478

[BIO059139C35] Sharma, N. and Venkadesan, M. (2022). Finger stability in precision grips. *Proc. Natl. Acad. Sci. U.S.A.* 119, e2122903119. 10.1073/pnas.2122903119 35294291 PMC8944252

[BIO059139C36] Steinberg, H. S., Winkle, T. V., Bell, J. S. and Lahunta, A. D. (2000). Cerebellar degeneration in old english sheepdogs. *J. Am. Vet. Med. Assoc.* 217, 1162-1165. 10.2460/javma.2000.217.1162 11043686

[BIO059139C37] Tabor, G., Elliott, A., Mann, N. and Williams, J. (2019). Equine posture analysis: development of a simple tool to record equine thoracolumbar posture. *J. Equine Vet. Sci* 73, 81-83. 10.1016/j.jevs.2018.11.011

[BIO059139C38] Thompson, P. D. (2012). Frontal lobe ataxia. *Handb. Clin. Neurol.* 103, 619-622. 10.1016/B978-0-444-51892-7.00044-9 21827922

[BIO059139C39] Valentine, B., De Lahunta, A., George, C., Summers, B., Cummings, J., Divers, T. and Mohammed, H. (1994). Article commentary: acquired equine motor neuron disease. *Vet. Pathol.* 31, 130-138. 10.1177/030098589403100122 8140721

